# Multifunctional Extracellular Matrix Hydrogel with Self-Healing Properties and Promoting Angiogenesis as an Immunoregulation Platform for Diabetic Wound Healing

**DOI:** 10.3390/gels9050381

**Published:** 2023-05-05

**Authors:** Zhenghua Sun, Hao Xiong, Tengfei Lou, Weixuan Liu, Yi Xu, Shiyang Yu, Hui Wang, Wanjun Liu, Liang Yang, Chao Zhou, Cunyi Fan

**Affiliations:** 1 Graduate School, Shanghai University of Traditional Chinese Medicine, 1200 Cailun Road, Shanghai 201203, China; 2 Department of Orthopedics, Shanghai Sixth People’s Hospital Affiliated to Shanghai Jiao Tong University School of Medicine, 600 Yishan Road, Shanghai 200233, Chinaly666@shsmu.edu.cn (L.Y.); 3 Shanghai Engineering Research Center for Orthopaedic Material Innovation and Tissue Regeneration, Building 3, Langu Science and Technology Park, Lane 70, Haiji 6th Road, Shanghai 201306, China

**Keywords:** quercetin, small intestine submucosa, hydrogel, wound healing, diabetic wound

## Abstract

Treating chronic wounds is a global challenge. In diabetes mellitus cases, long-time and excess inflammatory responses at the injury site may delay the healing of intractable wounds. Macrophage polarization (M1/M2 types) can be closely associated with inflammatory factor generation during wound healing. Quercetin (QCT) is an efficient agent against oxidation and fibrosis that promotes wound healing. It can also inhibit inflammatory responses by regulating M1-to-M2 macrophage polarization. However, its limited solubility, low bioavailability, and hydrophobicity are the main issues restricting its applicability in wound healing. The small intestinal submucosa (SIS) has also been widely studied for treating acute/chronic wounds. It is also being extensively researched as a suitable carrier for tissue regeneration. As an extracellular matrix, SIS can support angiogenesis, cell migration, and proliferation, offering growth factors involved in tissue formation signaling and assisting wound healing. We developed a series of promising biosafe novel diabetic wound repair hydrogel wound dressings with several effects, including self-healing properties, water absorption, and immunomodulatory effects. A full-thickness wound diabetic rat model was constructed for in vivo assessment of QCT@SIS hydrogel, in which hydrogels achieved a markedly increased wound repair rate. Their effect was determined by the promotion of the wound healing process, the thickness of granulation tissue, vascularization, and macrophage polarization during wound healing. At the same time, we injected the hydrogel subcutaneously into healthy rats to perform histological analyses of sections of the heart, spleen, liver, kidney, and lung. We then tested the biochemical index levels in serum to determine the biological safety of the QCT@SIS hydrogel. In this study, the developed SIS showed convergence of biological, mechanical, and wound-healing capabilities. Here, we focused on constructing a self-healing, water-absorbable, immunomodulatory, and biocompatible hydrogel as a synergistic treatment paradigm for diabetic wounds by gelling the SIS and loading QCT for slow drug release.

## 1. Introduction

Skin, the biggest body organ, is a barrier to viral and bacterial invasion. When a considerable area is affected, a decrease in skin integrity can result in severe illnesses or even death. The epithelial integrity disruption of the skin can result from surgical incisions or accidental injuries, including contusions, burns, lacerations, abrasions, and hematomas. Due to the critical effect of the skin on preserving homeostasis, its underlying normal tissues become dysfunctional, requiring speedy healing to restore skin continuity. 

A long-term effect of diabetes is a non-healing wound that is extremely painful for patients, both physically and emotionally. It places a heavy financial and social burden on society [1]. Diabetic wounds may be related to vascular dysfunction, peripheral neuropathy, decreased collagen production, and chronic inflammation. Additionally, chronic wounds have the features of increased expressions of pro-inflammatory factors, proteases, and reactive oxygen species. Unfortunately, existing treatments are not sufficiently efficient. Consequently, developing more efficient treatments to stimulate the healing of chronic diabetic wounds is necessary. Inflammation, hemostasis, remodeling, and proliferation represent four overlapping and synchronized processes of wound closure that occur normally [2]. Macrophages have been recognized as remarkable regulators of these processes. Growing evidence backs them because of their ease in changing their phenotypes in response to their received spatiotemporal stimuli in every phase [3,4]. These phenotypes have often been classified into two groups: pro-inflammatory M1 macrophages and pro-healing/anti-inflammatory M2 macrophages [5]. Macrophages usually exhibit phenotypic polarization from M1 to M2, thereby inhibiting acute injury. M2 macrophages can attract stem cells while secreting growth factors (GFs) and cytokines, thus, promoting the multiplication, differentiation, and migration of endothelial cells, fibroblasts, and keratinocytes [6]. Restoring the tissue microenvironment results in a positive feedback loop that promotes tissue regeneration by gradually increasing macrophage pro-healing activity [7]. It is related to angiogenesis, an essential process for oxygen and nutrient supply to cells to reconstitute the tissue matrix during remodeling [8].

Many types of wound dressings, such as solid particles, films, foam boards, and hydrogels, are crucial for accelerating chronic wound healing [9,10,11,12]. Hydrogel-based wound dressings have drawn much interest because of their high water content, softness, adhesiveness, and capacity to contain biochemical components, including cells and their derivatives, therapeutic ions, GFs, and miRNAs [13,14,15,16,17]. Additionally, developing extracellular matrix (ECM) materials combined with natural products as regeneration matrices or scaffolds is a vital research area [18,19,20]. The small intestinal submucosa (SIS) represents the decellularized ECM material obtained from the porcine jejunum. Its primary components consist of different collagens, glycosaminoglycans, adhesion molecules, and GFs, containing vascular endothelial GF, basic fibroblast GF, and transforming GF-b, and these are crucial for tissue regeneration [21,22,23,24]. The SIS is commonly used as a surgical mesh, cell culture matrix, wound dressing, tissue regeneration membrane, and tissue engineering scaffold because of its good biomedical applications, biocompatibility, hydrophilicity, and low immunogenicity [25,26,27]. To treat chronic wounds, various bioactive factors, such as nanoparticles, platelet-rich plasma, exosomes, and mesenchymal stem cells, are combined with the SIS hydrogel [20,28,29,30]. However, translating these techniques into clinical practice is still difficult due to high cost, a challenging delivery method, and possible negative effects of these encapsulated bioactive components. 

Quercetin (QCT) refers to a natural flavonoid that is extensively distributed in fruits and vegetables and generates powerful pharmacological effects against inflammation and free radicals [31]. According to previous studies, QCT may decrease the progression of degenerative conditions, such as atopic dermatitis, kidney fibrosis, periodontal inflammation, and osteoarthritis [32,33,34,35]. Moreover, recent studies have shown that it exerts broad pharmacological effects against oxidation, fibrosis, and tumors [36,37]. Importantly, QCT promotes fibroblast growth and inhibits scar generation and fibrosis, which may improve wound healing [38]. Simultaneously, it can regulate macrophages, thus, exerting an efficient anti-inflammation effect. A recent study showed that QCT could mitigate hepatic inflammation by suppressing M1 macrophage polarization [39]. Surprisingly, QCT could also promote protein kinase B phosphorylation, facilitating signal transducer and actuator of transcription 6 nuclear translocation, which later bound to genes associated with anti-inflammatory factors to increase M2-biomarker levels [33]. QCT is verified to decrease diabetic wound inflammation and pro-inflammatory factor levels (tumor necrosis factor-alpha [TNF-α] and interleukin [IL]-6) and to elevate anti-inflammatory factor levels (IL-10) and collagen distribution. Consequently, it can activate M2 macrophages and is promising in diabetic wound repair [40]. However, it is insoluble in water, and its ability to act on the skin is minimal. Therefore, to continuously provide QCT in local wounds, appropriate carriers, such as hydrogels, are warranted for ensuring a great loading rate and adhesive skin.

**Figure 1:**
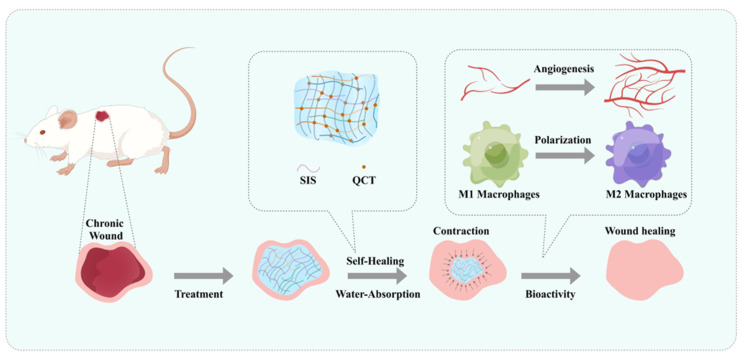
Schematic illustrating the synthesis, water absorption, self-healing, and bioactivity (angiogenesis and polarization) of the QCT@SIS hydrogel.

In the present study, a bioactive hydrogel containing ECM materials and natural flavonoids with antioxidant properties was prepared to regulate innate immunity and promote M1-to-M2 macrophage polarization. This hydrogel greatly promoted angiogenesis while activating the change in macrophage polarization to the anti-inflammatory and pro-healing subtype, thereby improving the anti-inflammatory effect and repairing the wound (Figure 1).

## 2. Results

### 2.1. QCT@SIS Hydrogel Characterization

**Figure 2:**
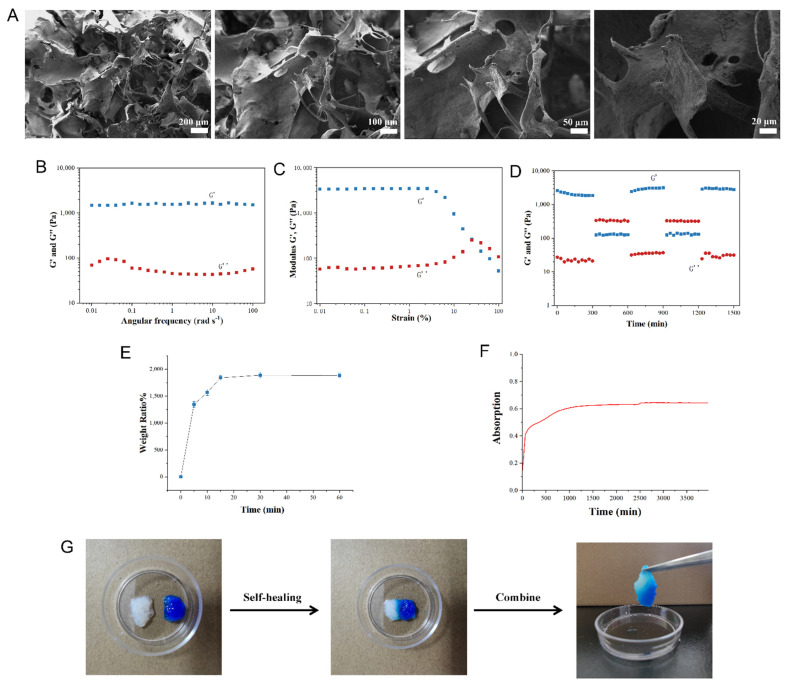
Morphologies, rheology, self-healing properties, and swelling ratio and drug release rate of the QCT@SIS hydrogel. (**A**) SEM images were used to observe the surface morphology in hydrogel samples at different magnifications; (**B**) oscillatory frequency sweeps of hydrogel in the fully swollen state; (**C**) strain amplitude sweep of hydrogel in the fully swollen state; (**D**) alternative step strain of hydrogel in the fully swollen state (strain 1% and 50%); (**E**) swelling ratios of hydrogel; (**F**) in vitro release profile of QCT by UV spectrometry; (**G**) macroscopic self-healing test of the QCT@SIS hydrogel in 10 min.

A scanning electron microscope (SEM) was adopted to observe the morphology of lyophilized QCT@SIS hydrogels, which exhibited an interconnecting porous structure (Figure 2A). Their pore size (20–500 µm) was randomly distributed, suggesting the presence of a continuous multistage pore architecture within the hydrogels, which allows cell migration and nutrient transport. After this, the rheological properties of QCT@SIS hydrogels were analyzed through dynamic rheology based on various parameters. When ω was between 0.01 and 100 rad s^−1^, QCT@SIS hydrogels retained a gel state, suggesting their stability (Figure 2B). Based on strain amplitude scanning analysis of QCT@SIS hydrogel, storage modulus (G′) and loss modulus (G″) curves intersected at the 26.5% strain level, suggesting the collapse of the hydrogel network at this point (Figure 2C). Additionally, to explore the self-healing ability of hydrogels, we performed various rheological recovery tests regarding alterations in the G′ and G″ of QCT@SIS hydrogels at a high strain level. Figure 2D presents the injury and recovery of the self-healing hydrogel at a high shear strain following healing at a low strain level separately. At the 50% dynamic strain level, G′ decreased approximately from 2572 Pa to 125 Pa in the hydrogels, resulting in G′ < G″, which indicated the collapse of the hydrogel network. At the 1% strain level, G′′ exhibited rapid recovery to the original level in QCT@SIS hydrogel, restoring its hydrogel structure. 

We then further analyzed the self-healing capacity of the hydrogel by macroscopic visualization. The separated parts of the hydrogels self-assembled into one part after 10 min of contraction without external force (Figure 2G), which might be related to the rich reconstruction of hydrogen bonds in SIS. In addition, the equilibrium swelling ratio of the QCT@SIS hydrogel was approximately 1889% (Figure 2E), which is much higher than that of commercial hydrogel materials. Higher swelling ratios help to maintain a moist wound environment and absorb tissue exudates; therefore, the QCT@SIS hydrogel is a suitable dressing for wound healing.

Additionally, an ultraviolet spectrophotometer was used to observe the drug release by hydrogel in phosphate buffer saline (PBS, pH = 7.4, simulated human normal tissue or blood). The absorbance value represented the change in the drug concentration in the solution. In addition, the drug concentration within the solution also gradually increased (Figure 2F), and the drug release reached equilibrium after 1000 min. At 4000 min, the drug release only increased slightly, indicating that the hydrogel could properly reduce the drug release rate.

### 2.2. Biocompatibility Test, Cell Migration, and In Vitro Angiogenesis

**Figure 3:**
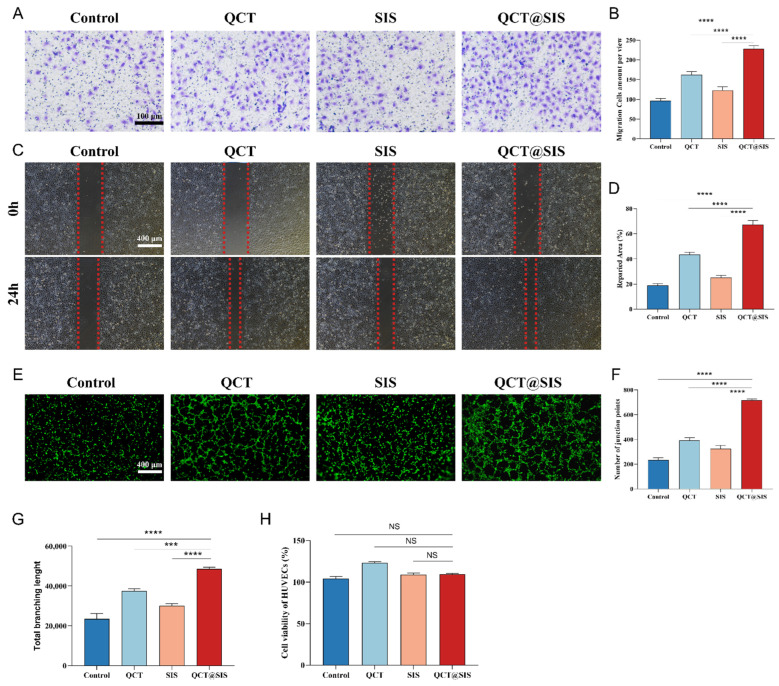
In vitro angiogenesis, migration, and biocompatibility evaluation of HUVECs induced by QCT@SIS hydrogel extract. (**A**,**B**) Transwell assay and quantification to detect the migration after 12 h with each sample (*n* = 3); (**C**,**D**) scratch assay and quantification to detect the migration after 24 h (*n* = 3); (**E**) representative fluorescent images of capillary tubes for 4 h incubation; (**F**,**G**) quantification of average junction points number and total branching length (*n* = 3); (**H**) cell viability for 12 h culture by CCK-8 assay (*n* = 3). Significant levels were determined at NS > 0.05, *** *p* < 0.001, and **** *p* < 0.001.

We evaluated the migration ability of the QCT@SIS hydrogel extract by transwell and scratch assays (Figure 3A,B). The average number of migrating human umbilical vein endothelial cells (HUVECs) in the QCT@SIS group in the transwell assay was 228. However, in the control, QCT, and SIS groups, the average migrating number of HUVECs was 96.33, 162.3, and 123.0. The number of migrating HUVECs in the SIS hydrogel group increased relative to that in the control group; however, they were still lower than that of the QCT@SIS hydrogel and QCT groups. This suggested that although the effect was insufficient, the SIS hydrogel extract certainly promoted cell migration. The cell migration-promoting ability of the QCT@SIS hydrogel extract was high relative to that of the other three groups. Figure 3C,D show the migration of HUVECs at 24 h by scratch assay. In the scratch test, the repaired area of the QCT@SIS group could reach 67.36%. However, in the control group, the QCT group, and the SIS group, this result was only 18.87%, 43.45%, and 25.27%, respectively. 

Consequently, the number of migrating cells in the QCT@SIS hydrogel group drastically increased compared with those in the other groups. Angiogenesis involves endothelial cell growth and tubular formation and migration, and the newly formed blood vessels delivering oxygen and nutrients to the wound site determine the diabetic wound healing outcomes [41]. We observed the angiogenesis-promoting abilities of HUVECs from each group in vitro by fluorescence microscopy (Figure 3E) and analyzed them quantitatively by the number of junction points (Figure 3F) and total branch length (Figure 3G). In the in vitro angiogenesis assay, the average junction points of the QCT@SIS group reached 716.3, while the total length of branches was 48,532. However, in the control, QCT and SIS groups, the average numbers of junction points were 234.3, 392.3, and 325.3, respectively. The total branching length also showed the same trend, with results of 23,527, 37,568, and 30,141, respectively. 

The analysis results revealed that the number of junction points in the QCT@SIS group increased relative to that of the control group. Moreover, compared with the SIS hydrogel and QCT groups, the QCT@SIS hydrogel group also had an increased number of junction points, and its effect on the in vitro angiogenesis-promoting ability was distinct. The analysis results of the total branching length were consistent with that of the junction points. The findings showed that QCT@SIS hydrogel positively affected the in vitro angiogenesis promotion in HUVECs, suggesting its remarkable wound-healing ability.

The Cell Counting Kit-8 (CCK-8) test was carried out to determine the vitality of HUVECs exposed to the hydrogel extract solutions for 12 h to assess the cytotoxicity of hydrogels. All the hydrogel extracts showed good biocompatibility. Cells cultured in the RPMI-1640 medium with no treatment were considered the control group. The cells grown in various hydrogel extract solutions presented no significant difference relative to the control group (Figure 3H). All the hydrogel extracts showed good biocompatibility in the in vitro experiments, indicating that HUVECs maintained high viability and proliferation ability throughout the procedure, regardless of QCT loading in the SIS hydrogel.

### 2.3. In Vivo Diabetic Wound Repair Tests

**Figure 4:**
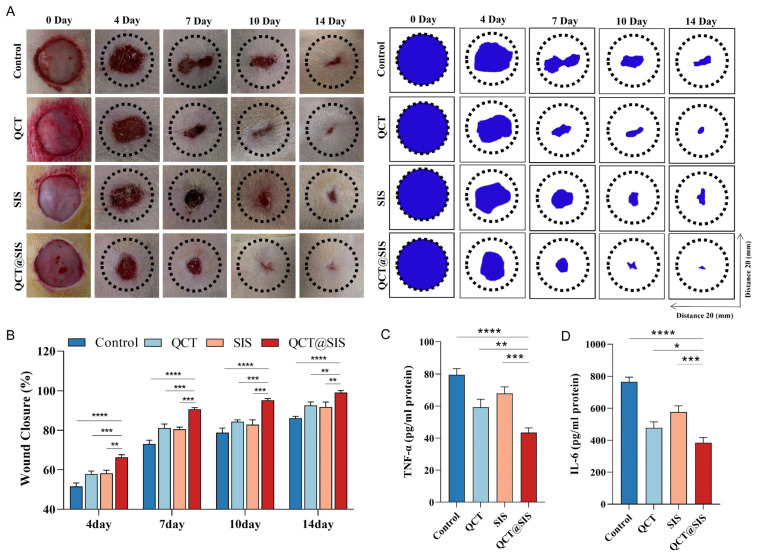
QCT@SIS hydrogel speeded up skin repair. (**A**) Representative images of wounds with different treatments and simulation diagram of the healing process on day 0, 4, 7, 10, 14 after wounding; (**B**) healing rate for each treatment (*n* = 3); (**C**,**D**) the release of cytokines in the wound following different treatments on day 14 after wounding (*n* = 3). Significant levels were determined at * *p* < 0.05, ** *p* < 0.01, *** *p* < 0.001, and **** *p* < 0.001.

To determine the role of QCT@SIS hydrogel in promoting skin wound healing, we used QCT@SIS hydrogel, SIS hydrogel, and QCT for full-thickness diabetic wounds, with the saline solution as a blank control. Figure 4A shows the size changes in skin wounds in the four groups on days 0, 4, 7, 10, and 14 post-surgery. We measured the extent of wound closure on days 4, 7, 10, and 14 using ImageJ software. Every treated wound significantly reduced in size on day 14, whereas the wound size in the control group decreased slowly during the experiment (Figure 4B). On day 14 after wounding, wound closure of rats in the QCT@SIS group averaged 99.132%, close to complete healing. Chen et al. [42] reported that chronic wound healing could be promoted by preparing mechanical SIS hydrogels. The mechanical SIS hydrogels healed 96.5% on day 11, while the QCT@SIS hydrogels reached 95.23% on day 10, further demonstrating the efficacy of QCT@SIS hydrogels. Similar to gross observations, the QCT@SIS hydrogel group had increased wound closure rates compared with those of the other groups throughout the healing process. Additionally, the healing impacts of the QCT@SIS hydrogel were considerably higher than SIS hydrogel, suggesting that the QCT@SIS hydrogel promoted wound repair by constantly releasing QCT. On day 14, skin tissues were collected and immersed in liquid nitrogen; then, tissue extracts were obtained for enzyme-linked immunosorbent assay (ELISA) analysis. We quantified the inflammation-related factors: TNF-α and IL-6 (Figure 4C,D). Thus, QCT@SIS hydrogel significantly decreased the levels of inflammation-related cytokines in rats. Parallelly, compared with QCT and SIS hydrogel, QCT@SIS hydrogel showed better therapeutic effects. Our findings conform to the previous reports. A previous study showed that QCT could accelerate the healing process by promoting macrophage polarization and reducing IL-6 and TNF-α in diabetic wounds [40]. Therefore, it can better promote the healing of diabetic wounds.

### 2.4. Histomorphological Evaluation

**Figure 5:**
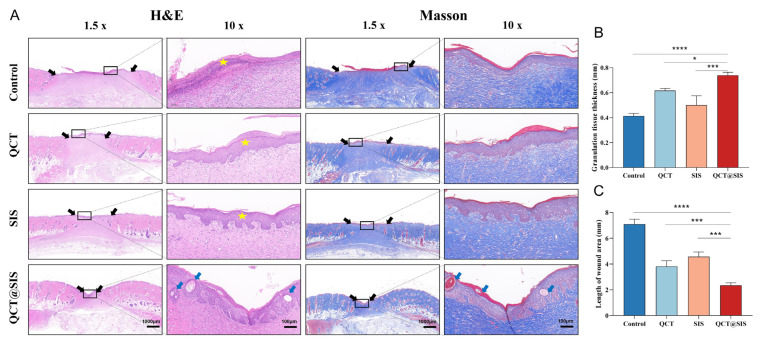
Histological analysis of skin tissue in diabetes wound for 14 day. (**A**) H&E and Masson staining of the wound sections. The black arrow demonstrate the length of wound area, the yellow stars demonstrate the remaining scars, and the blue arrow demonstrates the regenerated hair follicle.; (**B**,**C**) quantitative analyses of granulation tissue thickness and length of wound area (*n* = 3). Significant levels were determined at * *p* < 0.05, *** *p* < 0.001, and **** *p* < 0.001.

The effects of QCT@SIS gel on wound repair were compared with those of the QCT and SIS hydrogels, with saline solution serving as the control. On day 14, we harvested the wounded tissue for hematoxylin and eosin (H&E) and Masson staining to observe its structure (Figure 5A). Wounds in the QCT@SIS group were gradually altered to a volcanic shape, which is mostly associated with the wounds and surrounding new tissues. The wounds of each group gradually shrank over time. However, after 14 days of treatment, wounds in the QCT@SIS hydrogel group nearly closed and were accompanied by novel hair follicles, which had better repair than those in the other three groups. We visualized granulation tissue thickness and wound length in the pathological sections (Figure 5B,C). By histopathological analysis of the skin tissue on day 14 of the QCT@SIS group, the mean thickness of their granulation tissue was 0.739 mm and the mean length of their wound area was 2.344 mm.

Granulation tissue thickness varied among the four groups, which was significantly thicker in the QCT@SIS hydrogel group, suggesting that the SIS@QCT hydrogel can provide a suitable moist environment. SIS hydrogel can release various types of growth-promoting factors, and QCT can promote angiogenesis and inhibit inflammation by accelerating wound epithelial regeneration. Additionally, Masson staining results of the QCT@SIS hydrogel group indicated increased collagen fiber density and deposition within wounded tissues at day 14. The collagen fibers of the QCT@SIS hydrogel group were tightly and parallelly arranged, whereas those in the remaining three groups were loosely and irregularly arranged. A previous study showed that incorporating QCT into paraffin ointment could be used to treat diabetic wounds [43]. However, H&E staining demonstrated that the inflammatory infiltration of the wound tissue treatment by QCT paraffin ointment was near normal by day 21. In this study, the addition of QCT to the SIS gels normalized the wound tissue by day 14. This is due to the fact that SIS is a natural extracellular matrix material containing a variety of collagen, glycosaminoglycans, and growth factors, which together with QCT can exert a synergistic effect in the treatment of chronic wounds. Ma et al. [44] synthesized SIS hydrogels modified with extracellular vesicles to treat diabetic wounds. H&E staining showed that the skin tissue under this hydrogel treatment was significantly healed and covered the new neo-epidermis by day 14. Thus, the QCT@SIS hydrogel showed high-quality wound repair by promoting rapid epidermalization and increasing collagen deposition on the wound surface.

### 2.5. In Vivo Neovascularization Assessment

**Figure 6:**
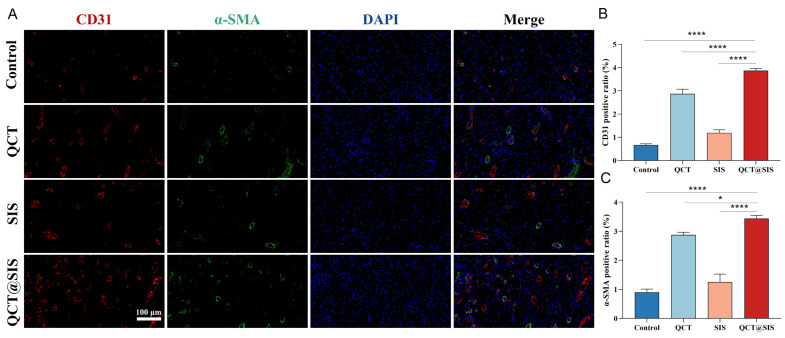
Immunofluorescence staining assay for angiogenesis. (**A**) Double immunofluorescence staining of neovascularization, CD31 structures (red) were surrounded by α-smooth muscle actin-positive cells (green) in different group; (**B**,**C**) quantitative analysis of the expressions of CD31 and α-SMA (*n* = 3). Significant levels were determined at * *p* < 0.05, and **** *p* < 0.001.

Neovascularization is a vital part of diabetic wound repair. Hyperglycemia in diabetes may cause vasoconstriction and inhibit angiogenesis, thereby hindering the wound healing process by blocking the oxygen supply. Angiogenesis plays a crucial role in the wound healing process, and it can deliver oxygen and nutrients to the wound area [45]. Based on in vitro analyses, QCT@SIS hydrogel had an effective angiogenesis-promoting effect on HUVECs. However, the in vitro angiogenic capacity of QCT@SIS hydrogel-affected angiogenesis in diabetic wounds is unknown. We evaluated the in vivo angiogenic effects of QCT@SIS hydrogel by immunofluorescent labeling of the angiogenesis marker cluster of differentiation-31 (CD31) and the vascular smooth muscle marker alpha-smooth muscle actin (α-SMA) [46,47,48]. Figure 6A shows the fluorescence of CD31 and α-SMA. The QCT@SIS hydrogel group exhibited better neovascularization. The average positive ratios of CD31 and α-SMA in the QCT@SIS group were 3.870% and 3.440%, respectively. The fluorescence analysis of CD31 and α-SMA demonstrated that the positive ratio significantly increased in the QCT@SIS hydrogel group compared with those in the remaining groups, with the pure QCT-treated group ranked second place (Figure 6B,C). Additionally, in the QCT@SIS hydrogel group, blood vessels exhibited larger size and luminal architecture, supporting the in vitro results of increased angiogenesis in HUVECs after the QCT@SIS hydrogel treatment. As reported in the previous study, diabetic wounds were treated by constructing a double cross-linked hydrogel. That the treatment of chronic wounds can be achieved by promoting neovascularization was shown by studying the expression of CD31 in skin tissue [49]. In another study, the authors treated diabetic wounds with a hydrogel prepared from oxidized dextran, platelet-rich plasma, and hyaluronic acid. High expression of α- SMA and CD31 was detected in the treated skin tissue [47]. In this study, we observed similar trends as in the previous study. This indicated the successful promotion of angiogenesis within the diabetic wounds by the QCT@SIS hydrogel.

### 2.6. Macrophage Polarization Assessment In Vivo

**Figure 7:**
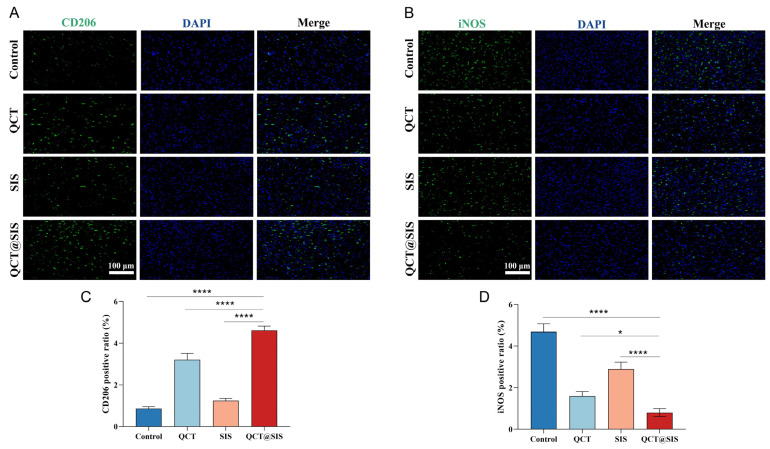
Immunofluorescence staining assay for macrophage polarization. (**A**,**B**) Representative images of immunofluorescence staining of iNOS and CD206 on day 14; (**C**,**D**) quantitative analysis of the expressions of iNOS and CD206 (*n* = 3). Significant levels were determined at * *p* < 0.05, and **** *p* < 0.001.

Changes in macrophage phenotype have an important effect on promoting wound repair, thereby requiring a pro-regenerative polarization of the M2 macrophages. As previously reported, QCT could reduce inflammatory infiltration by promoting proinflammatory M1 macrophage polarization toward M2 macrophages [50]. To explore the association between the QCT@SIS hydrogel-derived wound repair and alteration of macrophage polarization, we performed in vivo assays to understand macrophage activities. Based on the M1 macrophage marker inducible nitric oxide synthase (iNOS) and M2 macrophage marker cluster of differentiation-206 (CD206), we observed if QCT@SIS hydrogel could promote the polarization of macrophages in vivo (Figure 7A,B). Figure 7C,D show the results of the fluorescence quantification based on the analyses of the positive ratio of CD206 and iNOS. The average positive ratios of CD206 and iNOS in the QCT@SIS group were 4.613% and 0.797%, respectively. Compared with the pure SIS hydrogel group, CD206 expression in the QCT@SIS hydrogel group increased, whereas iNOS expression decreased. The pure QCT group promoted macrophage polarization, but the effect was not as good as that of the QCT@SIS group. The reason could be the slow QCT release by the QCT@SIS hydrogel to achieve a better therapeutic effect. The results showed that QCT@SIS hydrogel promoted polarization of the M2 macrophages.

Di Cristo et al. [51] synthesized a co-electrospun polylactic acid/poly(vinylpyrrolidone) fiber membrane as an adjustable QCT transport platform for diabetic wounds. Yang et al. [52] synthesized gallium-modified gelatin nanoparticles loaded with quercetin to promote skin wound healing. However, these studies were only validated in vitro and not in vivo. The results of the present study showed that the QCT@SIS hydrogel promoted the polarization of M2 macrophages through the in vivo release of QCT.

### 2.7. In Vivo Biosecurity Assessment

**Figure 8:**
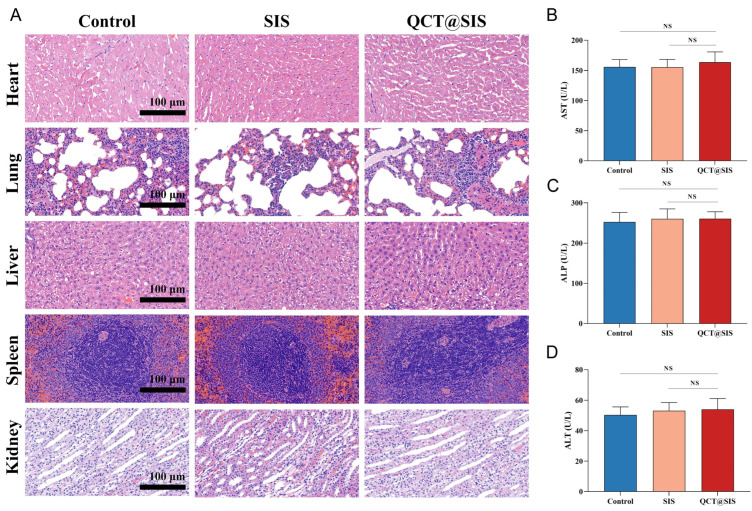
The biocompatibility of QCT@SIS hydrogel. (**A**) H&E staining of organs 14 days after injection into the shaved rat back; (**B**–**D**) Quantification of liver functional enzymes (ALT, ALP, and AST) in serum (*n* = 3). Significant levels were determined at NS > 0.05.

The biosecurity of hydrogel has always been a major challenge for clinical translation. We evaluated the biocompatibility of QCT@SIS through blood and histological analyses. To evaluate the biosecurity of hydrogel in vivo, we subcutaneously injected 100 µL hydrogel into healthy Sprague–Dawley (SD) rats. On day 14, the heart, spleen, liver, kidney, and lung of the rats were sectioned for H&E staining. The organ pathology of the rats remained almost unchanged (Figure 8A). Concurrently, we evaluated biochemical indexes, and the aminotransferase (AST), alkaline phosphatase (ALP), and alkaline phosphatase (ALT) levels in the QCT@SIS hydrogel group did not exhibit any significant differences compared with those in the control group (Figure 8B–D), suggesting that the QCT@SIS hydrogel group might not induce any acute or systemic blood and organ toxicities. Thus, QCT@SIS hydrogel showed good biocompatibility.

## 3. Discussion

With the development of a social economy, people pay more and more attention to the treatment of chronic wounds. Furthermore, the skin also features one of the most vulnerable tissues because of its direct contact with the outside world. Although most common skin lesions can largely return to their original appearance over time, skin lesions are difficult to restore 100% of skin function to. In addition, diabetic wounds do not undergo a normal healing process but rather fall into a chronic inflammatory phase featured by excessive accumulation of unrestrained M1 macrophages [53,54]. During healing, regulation of the release of cytokines, including TNF-α and IL-6, influences a variety of processes at the wound site, such as stimulation of fibroblast and keratinocyte proliferation, synthesis and degradation of fibroblast chemotaxis, extracellular matrix proteins, and modulation of immune responses [55,56]. However, excessive production of these cytokines and prolonged expression due to hyperactivation of immune cells may increase tissue destruction. Moreover, these cytokines trigger the production of proteases that degrade ECM [57,58]. Therefore, it is a novel idea to treat chronic diabetic wounds by promoting macrophage polarization and improving neovascularization in the wound skin.

As a biomaterial that can promote wound healing, SIS has been widely used as a bio-scaffold material in clinical use [59]. SIS, as an extracellular matrix, can support angiogenesis, cell migration, and proliferation, offering growth factors involved in tissue formation signaling and assisting wound healing [60]. In a clinical study using the SIS wound matrix for the treatment of chronic venous ulcers, a significant reduction in MMP-1, MMP-2, MMP-3, MMP-9, TNF-α, and IL-8 in wounds was observed [61]. Zhang et al. [62] loaded SIS membranes with silver nanoparticles, and the modified scaffolds effectively treated burn wounds infected with *Pseudomonas aeruginosa* with a reduction in IL -6 and C-reactive protein. Singh et al. [63] incorporated curcumin into SIS membranes. The modified membranes have good antimicrobial ability and can scavenge free radicals, effectively neutralizing oxidative stress and biofilm formation problems in skin wounds. SIS has, therefore, been widely recognized as an extracellular matrix biomaterial for its ability to repair wounds. In addition, the properties and functions of hydrogel—biocompatibility, biodegradability, low immunogenicity, extended treatment time, and ease of handling—make it more suitable for wound repair [64].

QCT is a bioflavonoid, with a strong antioxidant potential. Its antioxidant activity and anti-inflammatory properties to accelerate wound healing have been widely recognized [65]. QCT is a hydrophobic compound, and its poor water solubility and skin permeability hinder its activity [66]. Zhou et al. [67] developed an electrostatically spun chitosan/polycaprolactone nanofiber loaded with wound-healing compounds of rutin and QCT as an antimicrobial dressing. It has significant biocompatibility and antioxidant and bactericidal activity. Wangsawangrung et al. [68] developed a polyvinyl alcohol (PVA) hydrogel loaded with a QCT/cyclodextrin inclusion complex to improve the antioxidant activity and solubility of QCT. Wang et al. [69] prepared and investigated a multiphase hydrogel system containing liposomes loaded with quercetin for wound healing. The wound healing rate in rats increased significantly after the application of the hydrogel, and the wound decreased by approximately 90% after 2 weeks of treatment. However, conventional wound dressings are prone to damage during application to the skin. Therefore, in this study, we focused on developing self-healing hydrogels with customizable structures to improve the durability and reliability of hydrogels. 

In the present study, we synthesized SIS hydrogels by preparing SIS as a powder and prepared QCT@SIS hydrogels by incorporating QCT into SIS hydrogels. We constructed a diabetic rat model and applied QCT@SIS hydrogel to chronic wounds and showed that QCT@SIS hydrogel could significantly promote wound healing. With SEM, we observed that the freeze-dried SIS gels had a loose porous structure, which enabled better loading of QCT. In addition, rheological tests proved that QCT@SIS hydrogels have good stability and self-healing properties. The self-healing ability of QCT@SIS hydrogels may be their ability to have non-covalent interactions, dynamic covalent interactions and physical binding.Wound dressings are important to promote skin regeneration while resisting wear and tear from daily body movements [70]. Therefore, self-healing gels that are able to repair themselves after wound dressing injury are attractive. We measured the swelling properties of QCT@SIS hydrogels, and QCT@SIS hydrogels have good water absorption. Since wounds secrete a certain quantity of inflammatory secretions, hydrogels with good swelling properties are able to absorb the plasma secretions of the lesion and reduce the impairment of the wound healing process. Their role as a preferred wound dressing is widely accepted by clinicians and biomaterial scientists [70]. Ultraviolet spectrophotometry was used to measure QCT release rates in PBS, and the QCT@SIS hydrogels were able to release QCT continuously for 1500 min. Since the highly porous nature of hydrogels enables drug loading and release, this property can be easily adjusted by changing the cross-link density in their matrix structure [71]. 

In vitro, we performed CCK-8, migration and scratch assay. The results showed that QCT@SIS hydrogel could promote the migration of HUVECs. Tube formation experiments with HUVECs showed that QCT@SIS hydrogel can promote angiogenesis. Many studies have proved that impaired capillary remodeling and maturation may play a role in impaired diabetic wound healing [72]. In vivo, the wounds of diabetic rats in the group with QCT@SIS hydrogels were almost closed after 14 days. Moreover, histomorphological evaluation showed that QCT@SIS hydrogel promoted the formation of hair follicles and increased the thickness of granulation tissue. The skin tissue under this hydrogel treatment was well-healed and covered the new neo-epidermis. We demonstrated that QCT@SIS hydrogels can promote angiogenesis by immunofluorescent labeling of the angiogenic marker CD31 and the vascular smooth muscle α-SMA marker. Consistent with the in vitro results, QCT@SIS hydrogel could strongly promote neovascularization in diabetic wounds in vivo. Our study found that QCT@SIS hydrogels could inhibit the expression of M1 macrophage marker factor iNOS and promote the expression of M2 macrophage marker factor CD206 in vivo. In diabetic wounds, there are still M1 macrophages in the wound bed and almost no M2 macrophages [73]. This may lead to chronic inflammation and poor wound healing. Furthermore, this microenvironment of elevated glucose results in macrophages stimulating the secretion of proinflammatory cytokines, including IL-1ß, IL-6, and TNF-α, which encourages a vicious cycle of maintaining M1 macrophage polarization and chronic inflammation [74]. Thus, promoting polarization of M1 macrophages to M2 macrophages could treat diabetic wounds. Finally, we demonstrated the excellent biocompatibility of QCT@SIS hydrogels using histological sections of major organs and serum levels of AST, ALT, and ALP in rats. The results show that QCT@SIS hydrogels have good biocompatibility. This is due to the low immunogenicity of SIS as an extracellular matrix, and it also allows the slow release of QCT due to the porous adsorption properties of the hydrogel.

## 4. Conclusions

To conclude, in the current work, we demonstrated that the topical application of QCT can significantly accelerate diabetic wound healing. As an ECM material, SIS can also be used as a carrier for slow drug release. Prolonged inflammation due to an imbalance in macrophage polarization could delay diabetic wound repair. We developed a series of promising biosafe novel diabetic wound repair hydrogel wound dressings with several effects, including self-healing properties, water absorption, and immunomodulatory effects. A full-thickness wound diabetic rat model was constructed for in vivo assessment of QCT@SIS hydrogel, in which hydrogels achieved a markedly increased wound repair rate. Their effect was determined based on wound repair process promotion, granulation tissue thickness, vascularization, and macrophage polarization during wound healing. 

At the same time, we injected the hydrogel subcutaneously into healthy rats and performed histological analyzes of the heart, spleen, liver, kidney, and lung sections. We then tested the biochemical index levels in serum to determine the biological safety of the QCT@SIS hydrogel. In this study, the developed SIS showed a convergence of biological, mechanical, and wound-healing capabilities. Therefore, we believe that the QCT@SIS hydrogel could be widely used in regenerative medicine as a wound dressing, intelligent biomimetic soft material, and a substitute for medical devices.

## 5. Materials and Methods

### 5.1. SIS Membrane Fabrication

We prepared the native decellularized SIS membranes as previously described [75]. The serosa and muscle tissues were removed and rinsed with water six times. The SIS membrane was acquired from the small intestine of a pig and immersed in a methanol/chloroform (1:1 [*v*:*v*]) solution (Sinopharm Group, China) for 12 h at 4 °C with moderate stirring and then rinsed with PBS (Darthill Biotech, Shanghai, China) six times. SIS was then immersed in a mixture of 0.05% EDTA (Solarbio, Beijing, China) and 0.05% trypsin (Solarbio, Beijing, China) for a further 12 h while shaking gently. After washing with saline solution for 4 h, the SIS membrane was washed with 0.5% sodium dodecyl sulfate (SDS; Solarbio, Beijing, China) for 4 h at 4 °C while shaking gently. After a thorough water wash, the SIS membrane was retrieved and stored at 4 °C.

### 5.2. SIS Hydrogel Fabrication

SIS hydrogel was prepared as previously described [42,76,77]. The SIS membrane obtained in the previous stage was added to a solution of 0.5% acetic acid (Sigma-Aldrich, St. Louis, MO, USA) and slowly shaken for 12 h. Then, water was added to clean distended SIS membranes, followed by breaking them into small pieces using a barter machine. The temperature was lowered by adding an amount of cooled 0.5% acetic acid while breaking the SIS membrane. The small SIS chunks were removed and carefully shaken in trypsin solution (trypsin:SIS = 1:100 [*w*:*w*]) for two days at room temperature. After that, the solution was filtered through two layers of gauze to obtain a solution. NaCl (2 mol/L) was added to the solution after 24 h. Next, the solution was centrifuged for 15 min at 7800 rpm. In order to obtain the sediment, the solution was centrifuged four times. Sediments were thoroughly dissolved in 0.5% acetic acid before the salt extraction process was performed to obtain a more concentrated sediment. The SIS sediment was dissolved in glacial acetic before being transferred into a dialysis bag. PBS was added every two hours in the dialysis bag at 4 °C. The solution underwent three days of dialysis before being freeze-dried at 80 °C. A total of 1 *M* NaOH solution was then supplemented to adjust the pH of the SIS solution to 7.0. The solution was mixed with PBS (pH 7.0) under ambient temperature for 24 h to prepare 15% *w*/*v* SIS hydrogel.

### 5.3. Cell Culture

We obtained HUVECs from the Chinese Academy of Sciences Typical Culture Preservation Committee and cultivated them in the RPMI-1640 medium (Gibco, New York,USA) containing 1% penicillin–streptomycin (Solarbio, Beijing, China) and 10% fetal bovine serum (FBS) (Gibco, Thornton, New South Wales, Australia). The cells were incubated in a thermostatic incubator (Thermo Fisher Scientific, Waltham, MA, USA), including 5% CO_2_ at 37 °C.

### 5.4. Preparation and Characterization of the QCT@SIS Hydrogel

The preparation method of SIS hydrogel is mentioned in Section 2.2. To ensure an optimal drug loading rate to produce a QCT@SIS hydrogel, we added QCT (3.0 mg) to 5 mL SIS hydrogel. Later, a SEM (Hitachi S-4800, Tokyo, Japan) was employed to observe the QCT@SIS hydrogel microstructure. Briefly, after freeze-drying and gold-sputtering QCT@SIS hydrogel, a SEM was used for sample visualization at the 10 kV accelerating voltage. To determine QCT release behavior from QCT@SIS hydrogel, we introduced 1 mL of QCT@SIS hydrogel in the 15 mL tube, followed by the addition of 4 mL PBS in every tube before shaking at 100 rpm and 37 °C. Each sample was later examined in real-time for 4000 min. Ultraviolet spectrophotometry was performed to determine the QCT absorbance (OD) value at 374 nm. Finally, a plot regarding QCT cumulative release percentage was plotted after data processing. By measuring the initial drug amount (E_i_) and free drug after encapsulation (E_f_), we can determine the entrapment efficiency. Entrapment efficiency (EE) for QCT@SIS hydrogel was determined using the following formula:

$$\mathrm{Entrapment}\;\mathrm{efficiency}\;(\%)=\frac{E_{i}-{\;E}_{f}}{E_{i}}\times 100$$

The hydrogel rheological behaviors were analyzed using an AR-G2 rheometer (TA Instruments, New Castle, DE, USA). We used a rheometer with three oscillatory modes, namely time-, angular frequency-, and strain-dependent oscillatory modes. In the first oscillatory mode, the following parameters were used: ω was 10 rad s^−1^, the strain was 1% and 50%, the size of gel samples was 20 mm in diameter and 1 mm in height, and the samples were kept for 5 min at 1% or 50% strains. During measurements in angular frequency (ω) sweep test, gels (height, 1 mm; diameter, 20 mm) were kept at ω of 0.01−100 rad/s, and the strain was (γ) 1% at 25 °C. For measurements in the shear strain test, gels (1 mm high and 20 mm in diameter) were kept at a shear strain of 0.01−100% and a ω of 1% at 25 °C.

The swelling of the gel was measured at room temperature. SIS hydrogels were produced as previously described. The dried hydrogel sample, which had been pre-weighed, was submerged in a water-filled vial. At regular intervals, the hydrogel was taken out of the vial, the excess water was wiped off, and the hydrogel was weighed to determine weight change. Water was added back to the hydrogel. The weight change was noted until the weight stopped changing after three more measures. The swelling ratio was calculated as follows:

$$\mathrm{Swelling}\;\mathrm{ratio}\;\left(\%\right)=\frac{W_{f}-W_{i}}{W_{i}}\times 100$$

where W_i_ is the initial mass (dry sample) before suspension, and W_f_ is the maximum hydrogel mass.

### 5.5. Cytocompatibility Test for QCT@SIS Hydrogel

The SIS hydrogel and the QCT@SIS hydrogel were each submerged for 3 days in a cell culture medium without serum. The volume of the culture medium was increased by four times compared with that of the hydrogel. The SIS and QCT@SIS hydrogel extracts were obtained using a disposable 0.22 µm needle filter [78]. Based on previous studies, we used QCT (30 µg/mL) as a positive control group for in vitro experiments [79]. CCK-8 (Beyotime Biotechnology, Shanghai, China) was used to assess cell viability. Briefly, HUVECs (3 × 10^4^/well) were inoculated in 96-well plates for 12 h, followed by exposure to QCT and hydrogel extracts for 12 h. In addition, a microplate reader (Bio-Rad 680; Bio-Rad Laboratories Inc., Hercules, CA, USA) was used to measure OD values at 450 nm.

### 5.6. Migration, Proliferation, and Tube Formation Assays

A scratch assay was performed to determine cell migration. HUVECs were inoculated into 6-well plates at 1.2 × 10^5^/mL and cultivated to 100% confluence. The cells were later classified into four groups (control, SIS hydrogel, QCT, and QCT@SIS hydrogel groups). Parallel scratches were made with a 200 µL pipette tip in each well. Cells were then grown with various treatments for 0 and 24 h in an incubator with 5% CO_2_. The distance between the two scratches was observed under a light microscope (Leica, Wetzlar, Germany). With the use of Image J software (National Institutes of Health, Bethesda, Md.), original scratch (A_o_) and healing scratch (A_t_) ratios were computed. The following equation was adopted for calculating the migration ratio (A):

$$\mathrm{Migration}\;\mathrm{ratio}\;\left(\%\right)=\frac{A_{o}-A_{t}}{A_{o}}\times 100$$

We used a transwell system (Corning, New York, NY, USA) to perform a migration assay to assess the effect of QCT, SIS hydrogel, and QCT@SIS hydrogel on the proliferation of HUVECs. The lower chamber of the transwell system was filled with three intervention substances, whereas the upper chamber was filled with HUVECs (1 × 10^5^ cells/well). Three random fields were chosen for detecting migrating cells within 12 h. The 96-well plates were coated with 50 µL of Matrigel (BD Biosciences, Billerica, MA, USA) on ice for the in vitro tube formation assessment, followed by 45 min of incubation at 37 °C to promote gelation following specific protocols. After inoculating on Matrigel-coated plates, HUVECs (2 × 10^4^/well) were subjected to 4 h incubation in the blended medium (50 µL). After calcein-AM (0.002 mmol/L) staining, fluorescence microscopy was performed to visualize samples. Three random samples from every group were chosen to calculate the average junction point number and the overall branching length. 

### 5.7. Animal Model for Diabetic Wound

The animal experiments were performed using SD rats (12 weeks old, 250–350 g in weight). The experiments obtained approval from the Animal Research Committee of Shanghai Jiao Tong University Affiliated Sixth People’s Hospital. Streptozotocin (50 mg/kg; Sigma-Aldrich, St. Louis, MO, USA) was diluted with 0.1 M phosphate–citrate buffer (pH 4.5) and intraperitoneally injected into each rat to induce diabetes. Blood samples from the tail vein were obtained to identify the blood glucose (BG) levels using a glucometer (Roche, Indiana), and diabetes was diagnosed based on blood glucose levels of >300 mg/dL (16.7 mmol/L). Weight and blood glucose levels were checked twice a week.

Rats were given an intraperitoneal injection of 50 mg/kg pentobarbital sodium for anesthesia (*n* = 3 per group, PBS, QCT, SIS hydrogel, and QCT@SIS hydrogel). Later, we made wounds (diameter, 15 mm) with a full thickness on the dorsal skin using a surgical blade under aseptic circumstances after shaving and disinfecting. The PBS group and QCT group were treated with 1000 mL PBS or QCT (40 mg/mL). The drugs were added dropwise on the wounds gently until their complete absorption. The SIS hydrogel group and QCT@SIS hydrogel group were treated topically with 1000 µL of the hydrogels. Wounds of diabetic rats were intervened every 2 days during the entire 14-day treatment period. Photographs were taken from the same distance on days 7 and 14 following surgery to document the wound-healing process. After 14 days of wound formation, three animals from each group were sacrificed. The residual wound area (WA) was measured with the use of Image J software. The wound closure rate was determined as follows:

$$\mathrm{Wound}\;\mathrm{closure}\;\left(\%\right)\;=\;\frac{{\mathrm{WA}}_{4\mathrm{day}/7\mathrm{day}/10\mathrm{day}/14\mathrm{day}}}{{\mathrm{WA}}_{0\mathrm{day}}}\times 100$$

### 5.8. ELISA to Detect Inflammatory and Anti-Inflammatory Cytokines

The skin samples of each rat group were taken on day 14 and frozen in liquid nitrogen. Tissue extracts were extracted to perform an ELISA assay. Consistent with specific instructions, the expression of the associated inflammatory components was determined. RIPA lysis buffer containing 1 mM phenylmethylsulfonyl fluoride was added for sample homogenization with a tissue grinder, followed by 20 min centrifugation of homogenates at 12,500 rpm and 4 °C for collecting total protein. A rat ELISA kit (Affinity Biosciences, Changzhou, China) was used to determine TNF-α and IL-1β expression (pg/mg of protein).

### 5.9. Histopathology Analysis

Tissue samples were collected and gradually dehydrated, paraffin-embedded, and cut into 5 µm sections. Masson’s trichrome and H&E staining were carried out to visualize granulation tissue generation, epidermal regeneration, neovascularization, inflammatory cell infiltration, and collagen deposition.

We added 1.5% goat serum (Merck-Millipore) to the deparaffinized and rehydrated sections for blocking to perform immunofluorescence (IF) staining. Immunofluorescence staining was performed to detect the collagen arrangement, angiogenesis, and M1/M2 phenotypic transition of wound sections. Briefly, after sample dewaxing, primary antibodies against CD31 (Affinity Biosciences, Changzhou, China), α-SMA (Affinity Biosciences, Changzhou, China), iNOS (Affinity Biosciences, Changzhou, China), and CD206 (Santa Cruz Biotechnology, Santa Cruz, CA, USA) were added. Then, secondary antibodies were added, and nuclei were stained by DAPI (Sigma-Aldrich, St. Louis, MO, USA). A digital slide scanner was used to visualize the sections (Pannoramic MIDI, 3DHISTECH).

### 5.10. Biocompatibility of Hydrogels In Vivo

Each rat was given a subcutaneous injection of 100 µL of SIS hydrogel and 100 µL of QCT@SIS hydrogel on their back to test biocompatibility [80]. On day 14, vital organs, such as the kidney, lung, heart, spleen, and liver, were collected and stained by H&E. To determine hepatotoxicity, AST, ALP, and ALT activities in rat cardiac blood samples were measured concurrently. Blood was collected and centrifuged for 10 min at 6000× *g* to separate serum for blood biochemical analyses using associated kits (Kayto) through the automatic biochemical analyzer (Chemray 800) according to specific protocols.

### 5.11. Statistical Analysis

The mean and standard deviation of all statistical data are shown. Statistical analyses were performed using IBM SPSS Software (version 23.0; IBM, New York, NY, USA). To compare average values, one-way analysis of variance (ANOVA) with Tukey’s post-hoc test was performed. Significant levels were determined at * *p* < 0.05, ** *p* < 0.01, *** *p* < 0.001, and **** *p* < 0.001.

## Data Availability

Available upon request.
